# Development of a New Technique for Reconstruction of Hepatic Artery during Liver Transplantation in Sprague-Dawley Rat

**DOI:** 10.1371/journal.pone.0145662

**Published:** 2015-12-30

**Authors:** Xingmu Liu, Chao He, Tao Huang, Jiang Gu

**Affiliations:** 1 Guangdong Provincial Key Laboratory of Infectious Diseases and Molecular Immunopathology, Department of pathology; Collaborative and Creative Center, and Center of Translation Medicine, Shantou University Medical College, Shantou, Guangdong, China; 2 Department of General Surgery, Second affiliated Hospital, Shantou University Medical College, Shantou, Guangdong, China; UNIFESP Federal University of São Paulo, BRAZIL

## Abstract

**Background:**

Sleeve anastomosis is the most common technique used to rearterialize orthotopic liver transplants (OLT). However, this technique has a number of disadvantages, including difficulty of performance of the technique visually unaided. We herein describe a novel rearterialized OLT model in the rat.

**Materials and Methods:**

Forty-six male Sprague Dawley rats (300–400 g) were used as donors and recipients. Based on Kamada’s cuff technique, the new model involved performing a modified “sleeve” anastomosis between the celiac trunk of the donor and common hepatic artery of the recipient to reconstruct blood flow to the hepatic artery. An additional ten male Sprague Dawley rats underwent liver transplantation without artery reconstruction. Liver grafts were retrieved from the two groups and histological examination was performed following surgery.

**Results:**

Total mean operating times were ~42 minutes for the donor liver extraction and 57 minutes for the recipient transplantation. Graft preparation took an additional 15 minutes and the time to fix the arterial bracket was ~3 minutes. During transplantation, the anhepatic phase lasted 18 ± 2.5 min and the artery reconstruction only required ~3 minutes. The patency rate was 94.44% and the 4-week survival rate was 90%. Histology indicated obvious fibrosis in the liver grafts without artery reconstruction, while normal histology was observed in the arterialized graft.

**Conclusions:**

This new method allows for the surgical procedure to be performed visually unaided with good survival and patency rates and represents an alternative model investigating OLT in rats.

## Introduction

Lee and colleagues [1] developed the original technique for orthotopic liver transplantation (OLT) in the rat using a suture method to anastomose the liver vessels. Many investigators have sought to improve this surgical model of OLT due to the difficulties in performing the operation. Kamada and colleagues [2] introduced the “cuff” anastomosis technique for the portal vein (PV) and the infrahepatic vena cava (IHVC), which simplified the OLT procedure. Kamada’s surgical procedure did not include the reconstruction of the hepatic artery since it was well known that in the rat, rearterialization of the liver was not necessary for survival.

Nonarterialized OLT has been shown to be characterized by numerous liver defects, such as high rates of biliary complications [3]. Engerman and colleagues [4] recommended a rearterialized OLT model. Since both the donor common hepatic artery (CHA) and the recipient proper hepatic artery (PHA) are small, the anastomosis of the CHA and PHA are difficult. Engerman et al. achieved rearterialization by performing a end-to-side anastomosis between the donor aortic segment and the recipient infrarenal aorta [4].

Microvascular sleeve anastomosis was originially described by Lauritzen [5] and Duminy [6]. Subsequently, Howden [7] et al. successfully introduced an end-to-end sleeve anastomosis for OLT between the donor celiac axis and the recipient’s right renal artery. The sleeve technique greatly simplified arterial reconstruction during OLT, which improved the patency rate. However, in Howden’s method [7], the nephrectomy of the right kidney of the recipient was required. This model was greatly improved by the establishment of end-to-end anastomosis between donor CHA and PHA allowing for the preservation of physiological blood flow as described by Zhang [8] and Sato [9]. However, the task of performing the the sleeve technique in anastomose CHA and PHA is virtually impossible due to the small internal diameter (approximately 0.2–0.3 mm) of the PHA in a grown rat.

Therefore, the overall goal of the present study was to establish a novel method for anastomosis of CHA and PHA during OLT in the rat, which improves upon the current model by improving ease of the surgical procedure.

## Material and Methods

### Animals

Sprague Dawley rats (300–400 g), were obtained from the animal facility in the Medical College of Shantou University and were used as donors and recipients. The recipients were ~30 g heavier than the donors. Animals were housed in groups of four in temperature and humidity-controlled animal quarters and were allowed free access to food and water. All experiments were conducted under the guidelines and approval of the Medical Animal Care & Welfare Committee of Shantou University Medical College.

### Surgical Techniques

Donor surgeries were performed with pentobarbital anesthesia(2% pentobarbital, 0.3 ml/100g weight, ip) and recipient surgeries were performed with ether on cone mask (a cotton ball soaked in the exact amount of ether (2.75ml/l) was placed in a transparent jar with inhalation anesthesia. Prior to the procedure, recipients were treated with 0.1mg/kg atropine. Both surgical procedures were performed without an operating microscope and performed by a single investigator using clean, but not sterile surgical instruments. OLT was performed using a technique based on Kamada’s procedure [2] with the modification of a novel method to reconstruct the hepatic artery. By the end of designated period for each rat, it was sacrificed under ether anesthesia in transparent glass desiccator.

### Donor Operation

Following access of the abdominal cavity through a midline incision, the ligaments around the liver were dissected. The infrahepatic vena cava (IHVC) was freed before the right suprarenal vein and the right renal vein was ligated and carefully dissected. Subsequently, the bile duct was cannulated with a tube (0.8 mm OD, 5mm long) for biliary anastomosis, the portal vein (PV) was liberated, and the pyloric vein was ligated near the PV. The artery was liberated from the PHA to the celiac axis, and branches, such as splenic artery, left gastric artery, and gastroduodenal artery (GDA) were ligated and dissected. The liver was then perfused with cold physiological saline solution (4°C) containing heparin sodium (50 U/ml) via the PV and the suprahepatic vena cava (SHVC) was transected to release the perfusate from the liver. The liver was then quickly removed to a bath containing cold saline solution (4°C).

### Graft Preparation

**Fig 1:**
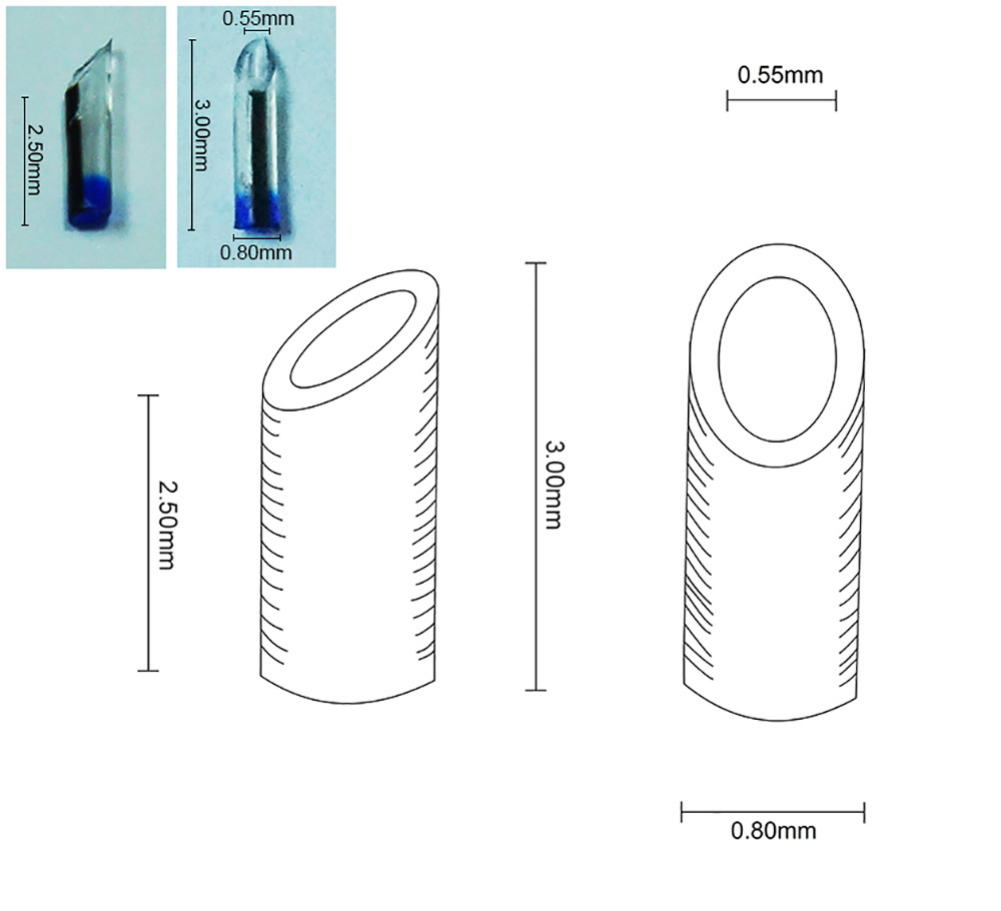
The tube that served as the bracket was made of polyethylene, and was designed as illustrated.

**Fig 2:**
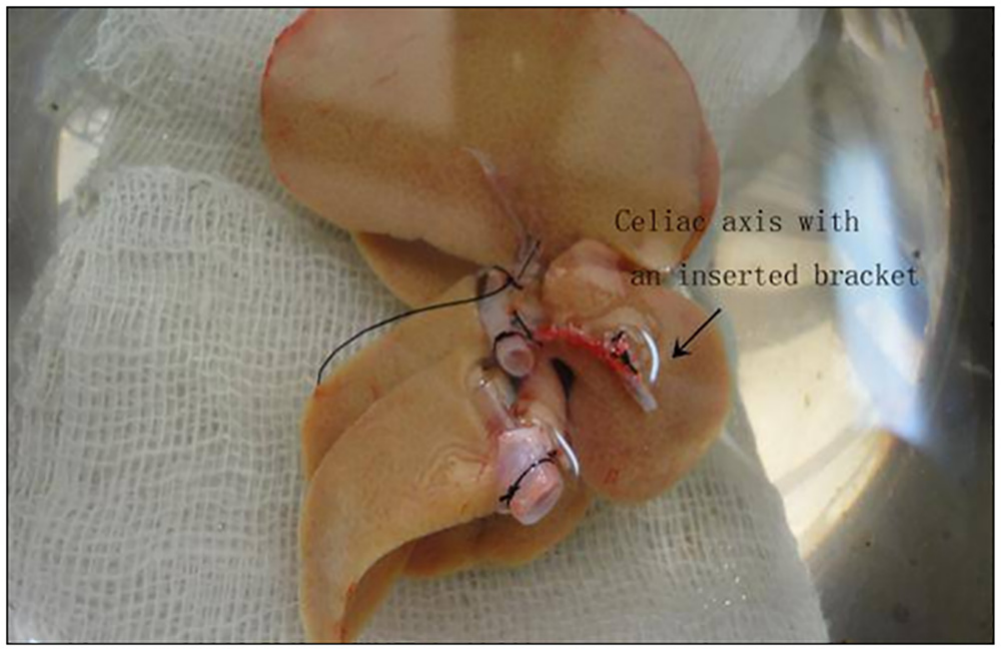
Donor liver was harvested and quickly placed in ice-cold saline. Preparation of the graft vessels was performed while submerged in saline.

Excess fat around the liver was trimmed, and then the blood vessels, cuff connection of the PV, and IHVC were constructed. The SHVC was trimmed carefully and attached with 8–0 double needle stitches on the left corner. The celiac axis was shaved and partially incised on the anterior wall. A sterile tube (Fig 1) that served as a bracket was then inserted into the celiac axis (Fig 2) and secured using a circumferential 8–0 silk ligature. Then the end of the celiac axis was carefully shaved.

### Recipient Operation

The recipients were anesthetized with ether and the abdominal cavity was accessed through a midline incision. The liver was liberated and the right suprarenal vein was ligated. After the bile duct and PHA were ligated and cut close to the hilum of the liver, the GDA was ligated at the beginning and transected. The CHA and stump of PHA were then liberated. The IHVC, PV, and SHVC were clamped in succession and were transected. Recipient livers were quickly removed. The donor liver was placed orthotopically in the recipient’s abdominal cavity. First, the SHVC was reconnected with a running suture (8–0 Proline) and then the PV was anastomosed using the cuff technique. The IVC anastomosis was performed using the same technique as with the PV after the blood flow of the liver was reestablished.

**Fig 3:**
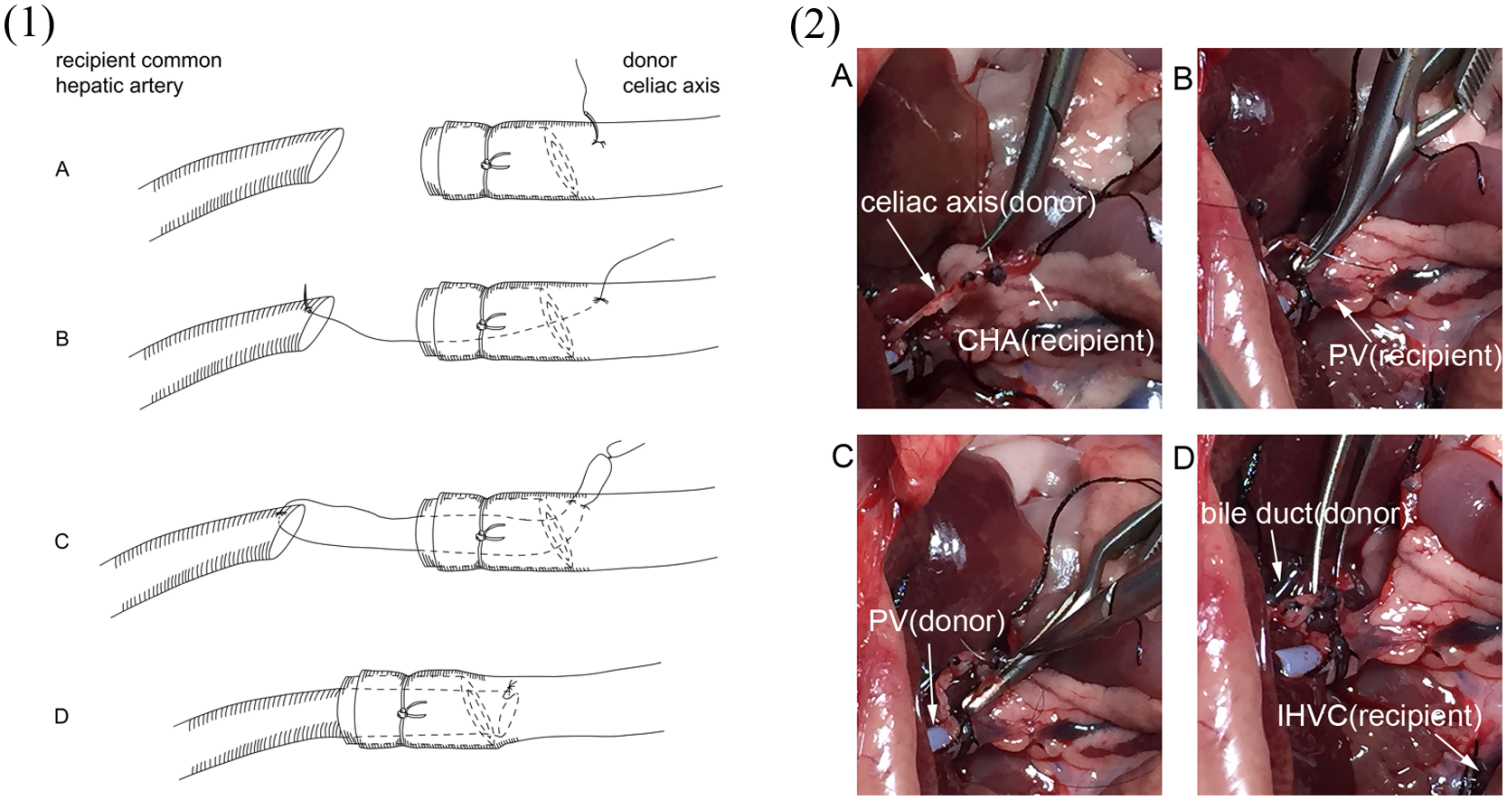
Depiction of the procedure of vessel anastomosis between donor celiac axis and recipient common hepatic(CHA). The vessel end of the recipient CHA and the donor celiac axis were placed closely to facilitate the anastomosis. (A): The first suture was performed with a length of 10–0 silk from outside to inside of the donor celiac axis and the suture was placed approximately 1 mm beyond the bracket. (B): The suture went transmurally through the anterior edge of the recipient CHA. (C): The donor celiac axis was repierced from inside to outside near the first suture point by the same thread. (D): Finally, the thread was pulled gently to guide the recipient CHA onto the donor celiac axis, and the suture was tied. Fig 3-(1) is diagram illustration showing the 4 steps of anastomosis. Fig 3-(2) shows the 4 steps with real photos of surgical procedure.

**Fig 4:**
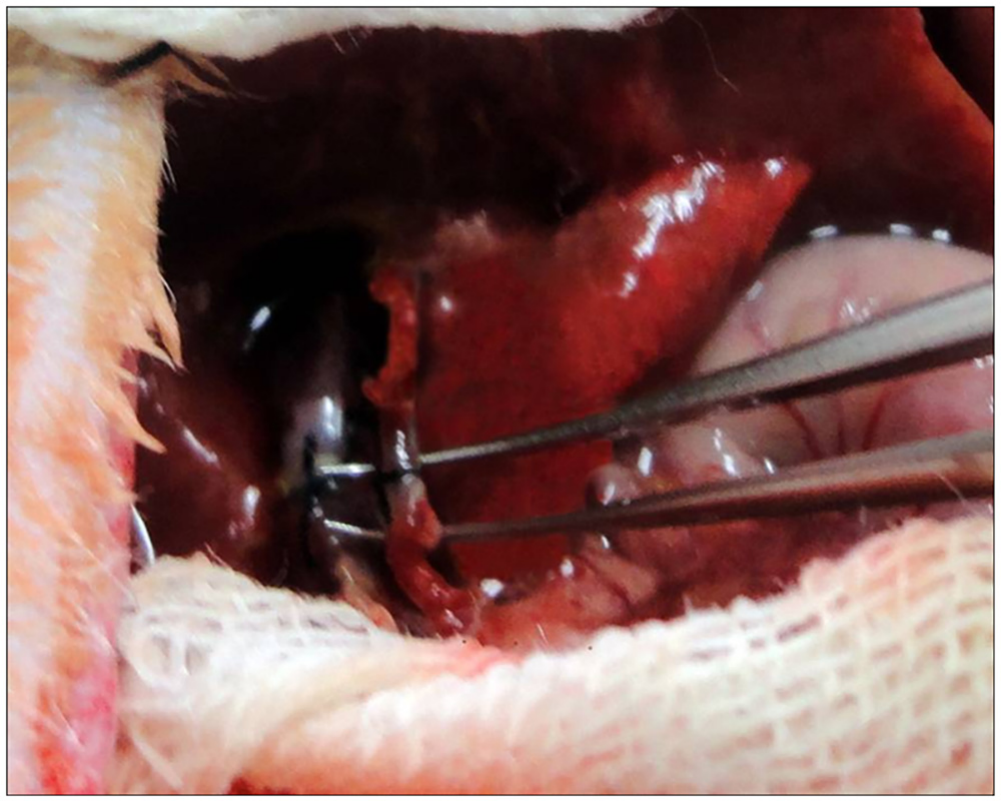
When the recipient CHA was unclamped, the vessel began filling and no bleeding was present at the site of anastomosis.

Reconstruction of the recipient hepatic artery was performed without the aid of an operating microscope. The recipient CHA was clamped at the beginning and transected at the end. The lumen of the vascular stump of both the donor and recipient were irrigated with saline solution. Loose adventitia of the recipient CHA was carefully removed up to the bifurcation of the celiac axis, while the adventitia of the donor celiac axis was gently removed from the first 2 mm of the stump. The vessel ends of both the donor and recipient were placed within a few millimeters. The guide suture pierced the vessel with 10–0 silk from outside to inside about 4 mm beyond the stump and about 1 mm beyond the bracket (Fig 3). The suture entered the lumen of the vessel, went through the bracket and out of the stump, and then the stitch went transmurally through the anterior edge of the recipient CHA. The stitch carefully went back into the lumen of the celiac axis and then pierced the vessel wall from inside to outside near the first suture point. Since the small bracket was inserted into the celiac axis to ensure that the vessel wall of the stump would not cave in, the operator could perform the guide suture placement easily and accurately. After the guide suture was in place, the thread of the suture was carefully pulled to guide the stump of the CHA into the lumen of the donor celiac axis, and slip through the bracket. The suture was then gently tied. When the vascular clamp was removed from the recipient CHA, the vessel began filling and no bleeding was found at the site of anastomosis (Fig 4). Vessels were inspected for patency by the presence of arterial engorgement and a positive filling test 5 minutes after completion of the anastomosis. After the HA reconstruction was completed, the recipient bile duct was incised on the anterior wall and the tube, which attached the donor bile duct, was tucked into the lumen of the recipient bile duct. The abdomen was closed using a running suture after the abdominal cavity was checked for hemostasis. After transplantation, the rats were allowed to recover from anesthesia in separate cages under an infrared lamp for half an hour, and subsequently returned to regular housing. Signs of distress and survival were monitored, during the first 12 hours post-transplantation rats were checked every 4 hours and subsequently every 8 hours for one week, and daily afterwards.

### Histology

Liver tissue was collected from the animal treatment groups. Tissues were fixed in paraformaldehyde, embedded in paraffin, and sections were cut and fixed on glass slides. Hematoxylin-eosin staining was performed to check the tissue histology.

## Results

In the current study, 23 cases of rearterialized OLT were performed using a novel anastomosis technique. All of the rats survived up to 4 weeks with the exception of two that died within 10 days post-transplantation. One cause of death was due to blood leakage at the hepatic artery anastomosis (On autopsy of the died rat, it was found that there was old blood clot arround the hepatic arterial suture. When the clot was removed, we observed that the anastomotic ligation was not tight following rinsing with water. We believe that this might have caused the loose knot.) and the other was due to portal vein stenosis. The survival rate at 4 weeks post-transplantation was 91.3%(21/23).

Total mean operating time was ~42 minutes for donor operation and 57 minutes for recipient operation. The graft preparation time was 15 minutes and the operating time to fix the arterial bracket was ~3 minutes. During the recipient operation, the anhepatic phase lasted 18 ± 2.5 min and the arterial reconstruction only required ~3 minutes.

Four weeks after OLT abdominal exploration was performed prior to the sacrifice of the animals in the arterial reconstruction group with the exception of 3 cases that were evaluated 6 months following transplantation. The hepatic artery was dissected clearly to make sure that the arterial pulsation above the site of artery anastomosis was apparent. The arterial branches near the liver hilus were then cut, and blood flowed immediately. This confirmed the patency of the reconstructed artery. We observed in only one case that the reconstructed artery was not present in the abdomen of the recipient 4 weeks following transplantation. In this case, occlusion might have occurred at the site of the artery anastomosis, making the occluded artery difficult to distinguish from the surrounding tissue because of inflammatory adhesions. The patency rate of the reconstructed artery was 94.44% (17/18), which is similar to rates reported in other studies [10].

**Fig 5:**
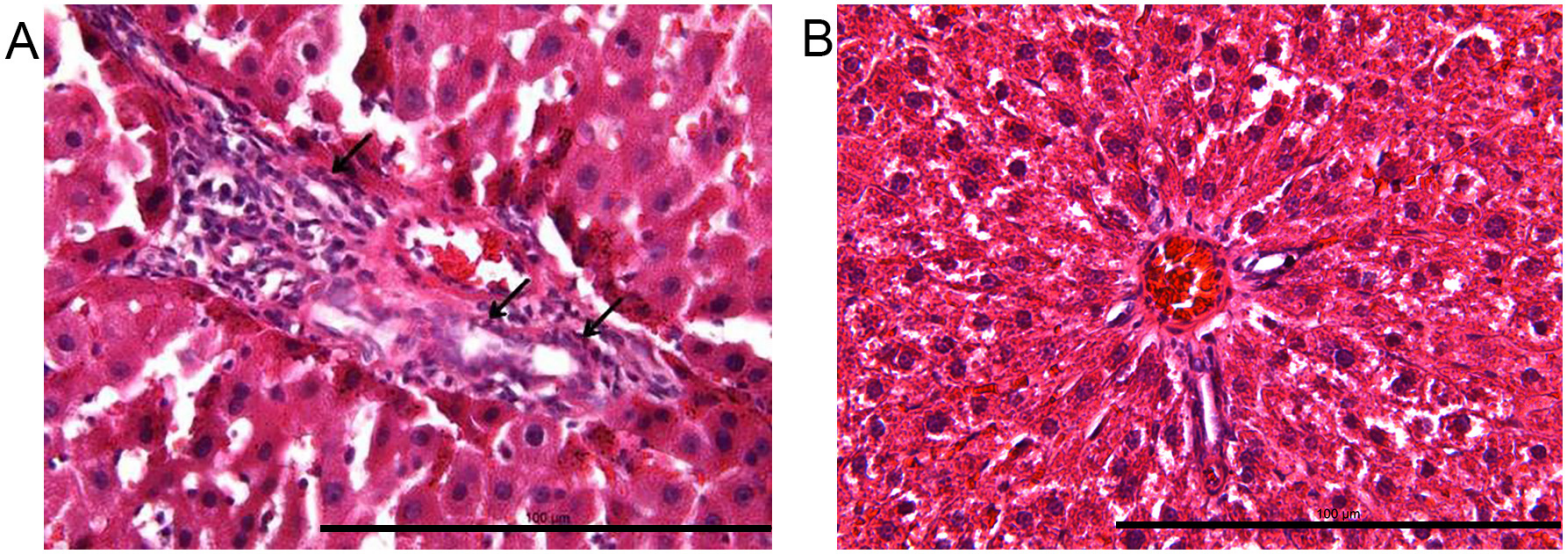
Histology in the nonarterialized graft. (A) Moderate biliary proliferation was observed as indicated by the arrow. (B) No obvious morphological changes were observed in the arterialized graft and the portal tracts were of normal size and no biliary proliferation was present.

In the group of animals that were sacrificed 6 months after OLT, obvious liver histomorphological changes were observed in animals without arterial reconstruction such as the proliferation of the biliary system (Fig 5).

## Discussion

We found that ether anesthesia is more convenient and the induction period is shorter than those of the other methods. The dosage could be adjusted according to the depth of anesthesia, when the operation is completed, and the anesthesia is disconnected, rats wake up almost immediately. Therefore, we chose ether as the anesthesia for our experiment. Due to the high survival rate following surgery, the nonrearterialized model of OLT (NOLT) in the rat is commonly utilized. However, it has been shown that NOLT models have several disadvantages: (i) a high rate of biliary complications [11]; (ii) lack of hepatic artery infusion, resulting in a liver that is physiologically different than the pre-transplantation livers [12]; (iii) hypoxia in the graft due to long-term vascular support from only venous circulation [13–15]; and (iv) lobular inflammatory infiltrate due to liver damage [16–18]. In addition, the phenomenon of hepatic ischemia/reperfusion (I/R) injury occurs during OLT [19]. After liver transplantation, patchy or/and focal necrosis is an important histopathologic finding in I/R injury [20]. Most of the blood to the bile duct in a transplanted liver is supplied by the hepatic arterial system [21]. It is an independent risk factor for an ischemic-type biliary lesion and lethal biliary complications due to poor blood supply by the hepatic artery in the early stage following liver transplantation. In Fig 5, proliferation of the biliary system was observed after OLT without arterial reconstruction, which may be associated with no blood supply by the hepatic artery in the early stage. Insufficient blood supply dramatically increases the hepatic I/R injury. Therefore, hepatic rearterialization after OLT protects the bile duct and reduces hepatic I/R injury. In consequence, rearterialized OLT (AOLT) should provide a more ideal model that can be used for transplantation research studies.

Several methods have been developed for liver transplantation in the rat [22–26], Recently, these methods have been modified to include hepatic artery rearterialization. The original and standard AOLT model in the rat is performed by an end-to-side anastomosis between the donor’s and recipient’s abdominal aortas by Engerman et al [4]. Since this method involves the clamping the recipient’s infrarenal abdominal aorta during the arterial anastomosis, there is a period of ischemia in all organs below the kidneys, which makes this method a less optimal approach.

Duminy introduced a new sleeve technique especially for microvascular anastomosis [6]. As Duminy [6] described, a sleeve anastomosis is placed at the end of the feeding vessel into the end of the receiving vessel. Recently, Howden [7] and colleagues introduced this technique for the reconstruction of the hepatic artery. Various of modifications have been made to reduce the difficulty, decrease the operation time and maintain a high patency rate of the hepatic artery. The sizes of the feeding and receiving vessels greatly influence the difficulty of the anastomosis. The sleeve technique was initially used in OLT for the anastomosis the donor’s celiac axis and the recipien’s right renal artery in AOLT by Howden [7] et al. Recently, Liu [27] performed the sleeve anastomosis between the recipient celiac axis and donor celiac axis. Both of these studies reported high patency rates. However, with these methods right nephrectomy or the complete ligation of the splenic, left gastric artery are unavoidable.

In addition, when the sleeve anastomosis is performed as described above, the guiding stitches are normally placeed outside to inside 2–3 mm beyond the stump. Even if the donor celiac axis has been chosen for the placement of the guiding stitches, the diameter is near 1 mm without blood flow, which makes the technique difficult to learn for inexperienced investigator or for a single operator to place the guiding stitches accurately in such a small vessel. Technically, the placement of the guiding stitches are the most important component of the sleeve anastomosis and technical difficulties are increased when the diameter is small.

**Fig 6:**
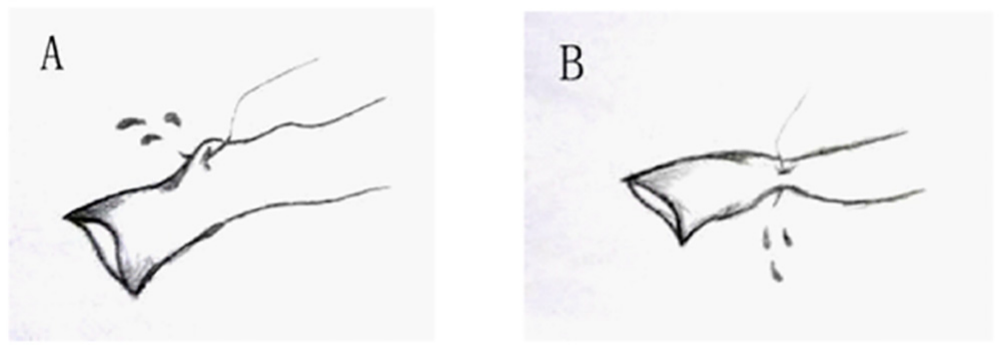
Illustrations of potential accidents during the placement of the guiding stitches.

As illustrated in Fig 6, there may be an increased incidence of surgical errors when the surgeon is initially learning the technique for the placement of the guiding stitches in small diameter vessels. These issues concerning the accurate placement of the first suture were worked out by trial and error prior to the start of our study. We found that the use of a bracket inserted into the celiac axis, reduced and in most cases eliminated surgical errors during the placement of the guiding stiches.

In the current study, anastomosis was performed between the donor celiac artery and the recipient CHA. The bracket ensures that the opening of the vessel does not collapse, facilitating the placement of a guide suture, which can be performed accurately and easily by a single operator without the use of an operating microscope. In addition, when the celiac artery is fixed with a bracket, the inside diameter of the vessel constricts to a size that is close to that of the recipient CHA, making the anastomosis between the two arteries possible despite the large difference in the diameters of the two vessels. Most importantly, nephrectomy or the complete ligation of the splenic, left gastric artery is avoided because the recipient’s renal artery or celiac axis is not required for anastomosis. Finally, when blood flow is restored, the vessel wall of the CHA is braced by the bracket, which avoids blood leakage. In the trial tests prior to the start of the current study, external diameter and internal diameter of the bracket was designed at1 mm and 0.75 mm, respectively. When performing the artery anastomosis, bleeding was often encountered at the anastomotic site. Therefore, the bracket was modified until bleeding was not found after artery anastomosis while ensuring a high patency rate.

In Liu’s [27] and Howden’s [7] studies, the time of artery anastomosis was reported to be approximately 10 minutes. In the current study, the time of arterial anastomosis was significantly reduced. In addition, since nephrectomy or the preparation of the celiac axis was not required during the recipient’s surgery, the recipient operation time was also reduced in the current study, which may lessen the probability of infection and other complications.

During graft preparation, the bracket was inserted into the graft celiac artery without difficulty. Since the operation was performed in cold saline solution, the natural expansion of the stump facilitated bracket insertion. Although the bracket might cause rejection in the rat resulting in thrombosis at the site of artery anastomosis, no major issues were observed and the liver patency rate was similar to that of other reported techniques. With our modified method, the bracket was sandwiched between two layers of the vessel wall and not exposed to blood, thereby reducing the possibility of thrombosis and ensuring a high patency rate. In our experiments, 4 weeks after surgery, the hepatic artery was dissected to assure that the arterial pulsation above the site of artery anastomosis was apparent. The arterial branches near the liver hilus were then cut, and blood flowed immediately. This confirmed the patency of the reconstructed artery. Lu et al [28], also confirmed the hepatic artery patency with the same approach. Overall, the animals recovered quickly following surgery and no significant changes in histology were observed in the grafted liver, which is in contrast to the non-arterialized method.

In general, the modified sleeve technique in reconstruction of the HA described here has proven to be both feasible and practical. The benefits of our approaches included rearterialization surgery without using a microscope, no need for recipient nephrectomy, and no interruption of perfusion to other abdominal organs. The patency rate of reconstructed HA was similar to other methods and was characterized by few complications during the 4 weeks follow-up period. Our method represents a significant improvement of the previous models and provides for physiological transplantation of the liver. This new, simplified surgical approach may provide an additional surgical option for scientists interested in transplantation research.

## Abbreviations

OLT: orthotopic liver transplants
PV: portal vein
IHVC: infrahepatic vena cava
CHA: common hepatic artery
PHA: proper hepatic artery
GDA: gastroduodenal artery
SHVC: suprahepatic vena cava
NOLT: nonrearterialized model of orthotopic liver transplants
AOLT: rearterialized orthotopic liver transplants
I/R: ischemia/reperfusion
